# Regenerative Endodontics-Reviving the Pulp the Natural Way: A Case Report

**DOI:** 10.7759/cureus.36587

**Published:** 2023-03-23

**Authors:** Simran Das, Rashi Srivastava, Nilima R Thosar, Monika Khubchandani, Rutuja Ragit, Nishi Malviya

**Affiliations:** 1 Pediatric Dentistry, Sharad Pawar Dental College, Datta Meghe Institute of Higher Education and Research, Wardha, IND

**Keywords:** triple antibiotic paste, mineral trioxide aggregrate, platelet-rich fibrin, immature permanent teeth, regenerative endodontics

## Abstract

Regenerative endodontic therapy (RET) offers a novel treatment option for developing teeth with pulp necrosis. In the current instance, RET was used to treat an immature mandibular permanent first molar that had been identified with irreversible pulpitis. The root canals were treated with triple antibiotic paste (TAP) and 1.5% sodium hypochlorite (NaOCl) irrigation. TAP was removed, and 17% ethylenediaminetetraacetic acid (EDTA) was used to treat the root canals during the second visit. As a scaffold, Platelet-rich fibrin (PRF) was applied. Mineral trioxide aggregate (MTA) was applied over PRF, and composite resin was used to repair the teeth. Radiographs taken from the posterior were utilized to assess the healing. The teeth displayed no signs of pain and healing after the six-month follow-up periods, and pulp sensibility tests using a cold and electric pulp tester produced no results. Conservative treatment options should be considered to save immature permanent teeth and assist in the regeneration of the root apex.

## Introduction

Immature permanent teeth have traditionally been challenging to treat endodontically. Two approaches are commonly used: apexogenesis, which involves keeping the pulp tissue alive and allowing the root to form naturally, and apexification, a procedure in which the appropriate materials are used to form a barrier and close the apex of the root. The apexification approach has many drawbacks. Repeated intracanal medication placement, which increases the risk of reinfection, is required when calcium hydroxide is used, and extensive instrumentation inside the root canal may weaken the canal walls [1,2]. Replacing intracanal medication is solved using novel materials that can replace calcium hydroxide, such as Mineral trioxide aggregate (MTA) or Biodentine. None of these materials, however, promote root completion, and they only serve as a mechanical barrier that closes the lumen of the root canal [3].

Regenerative endodontic therapy (RET) utilizes biological and engineering principles to restore the pulp-dentine complex, which various factors like caries, traumatic injury, or abnormalities have harmed. It has been suggested as the optimal course of treatment for young permanent teeth with necrotic pulp and apical periodontitis [4]. The outcomes should be similar to those of apexogenesis, which is an essential process in treating vital pulp [4].

A blood clot within the root canal system acts as a biological scaffold, producing successful results in most RET [5,6]. Other forms of physical scaffolds have also been known to generate tissue regeneration. The second-generation platelet concentrates, or platelet-rich fibrin (PRF), contains platelets, growth factors, and cytokines that continuously improve the healing capability of the teeth. PRF is completely autologous in origin [7,8]. This case report details a regenerative endodontic procedure for a mandibular right first molar with symptomatic irreversible pulpitis employing triple antibiotic paste, MTA, and PRF.

## Case presentation

**Figure 1:**
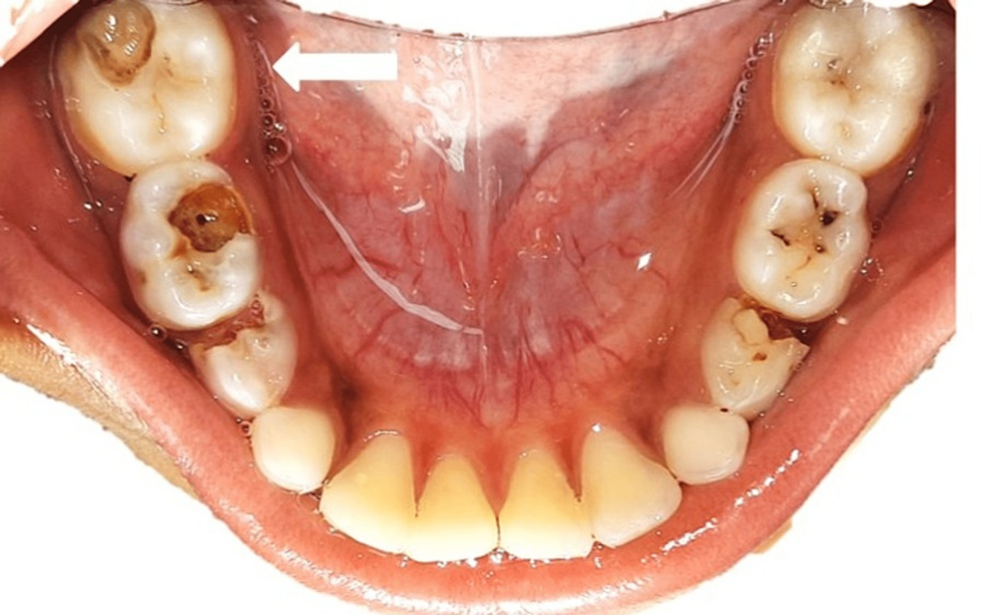
Pre-operative intraoral image Deep occlusal caries associated with the lower right mandibular first molar can be seen (White arrow).

**Figure 2:**
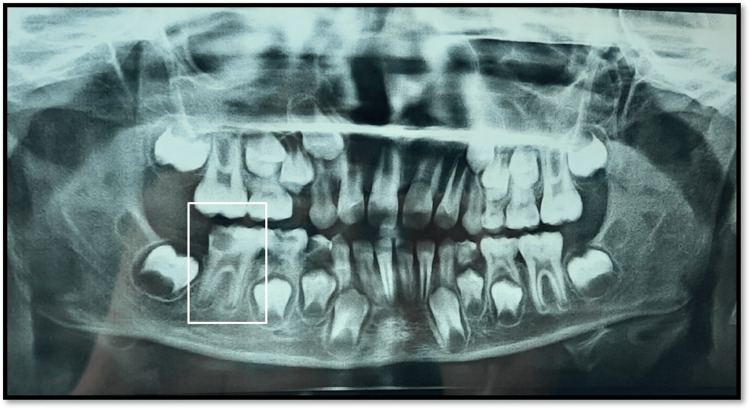
Pre-operative radiograph Orthopantogram (OPG) shows incomplete root formation and increased periodontal ligament space with mandibular right first molar (White box).

A 7-year-old male patient reported to the Department of Pediatric And Preventive Dentistry with a chief complaint of pain in the lower right back region of the jaw for 20 days. The patient was all right 20 days back when he started experiencing pain in the jaw's lower right back tooth region. The pain was sudden onset, moderate to severe in intensity, localized, and sharpshooting. The medical and dental history was non-contributory. On intra-oral examination, the right first permanent molar had deep occlusal caries tender on percussion (Figure 1). Radiographically, incomplete root formation and increased periodontal ligament space were seen in the mandibular right first molar (Figure 2).

A diagnosis of symptomatic irreversible pulpitis with open apex was made. RET with the mandibular right first molar was considered the best course of action considering all the factors. Parental consent was acquired after thoroughly explaining the complications and alternative treatment options.

**Figure 3:**
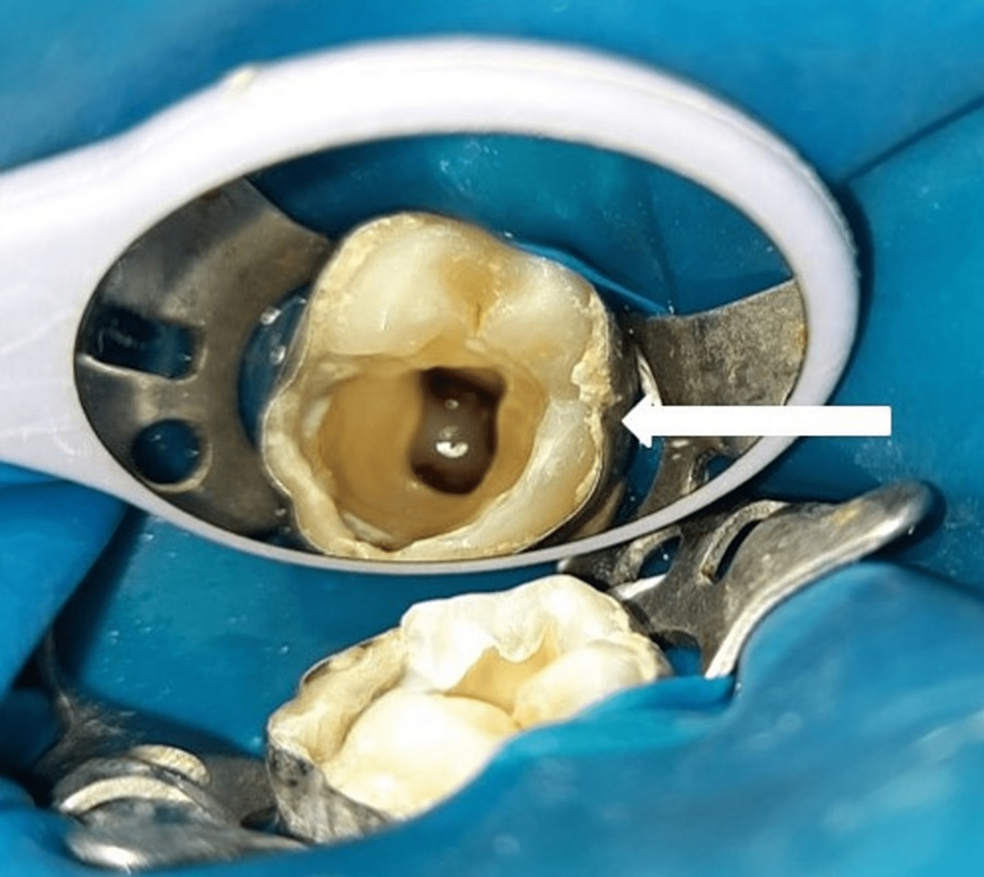
Access opening done followed by copious and gentle irrigation with 20ml 1.5 % Sodium hypochlorite (White arrow)

The RET was carried out in two appointments. In the first appointment, local anesthesia was administered, and dental dam isolation and access were gained. Since the tooth was affected with deep occlusal and disto-proximal caries, no distal wall remained after the caries excavation. The distal wall buildup was done with composite, followed by banding to support clamp placement during rubber dam placement. After locating all the canals and pulp extirpation, copious and gentle irrigation with 20ml of 1.5% Sodium hypochlorite (NaOCl) was done (Figure 3).

To reduce cytotoxicity to stem cells in the apical tissues, saline irrigation with an irrigation needle around 1mm from the root end was performed. Working length determination was done. The canals were dried with paper points. A thin layer of triple antibiotic paste with a final concentration of 0.1-1.0 mg/ml was applied over the canals in a 1:1:1 ratio of ciprofloxacin, metronidazole, and minocycline. The pulp chamber was sealed with a dentin bonding agent to minimize the risk of staining before the triple antibiotic paste was placed. The tooth was sealed with 3-4mm glass ionomer cement as a temporary restorative material. The patient was after 4 weeks for the second appointment.

There was no indication of a persistent infection when the effectiveness of the initial treatment was evaluated at the second session. Ten ml of blood was drawn from the patient's left forearm and centrifuged at 2700 rpm for 12 minutes to extract the PRF in the membrane form. Copious, gentle irrigation with 20 ml of 17% ethylenediaminetetraacetic acid (EDTA) was done in all the canals. PRF membrane was cut into small strips, placed, and condensed in each canal. Bleeding was stopped at a point where 3-4 mm of restorative material could be inserted. The capping material was white MTA. The final composite restoration was completed over a 3-4 mm layer of glass ionomer cement.

**Figure 4:**
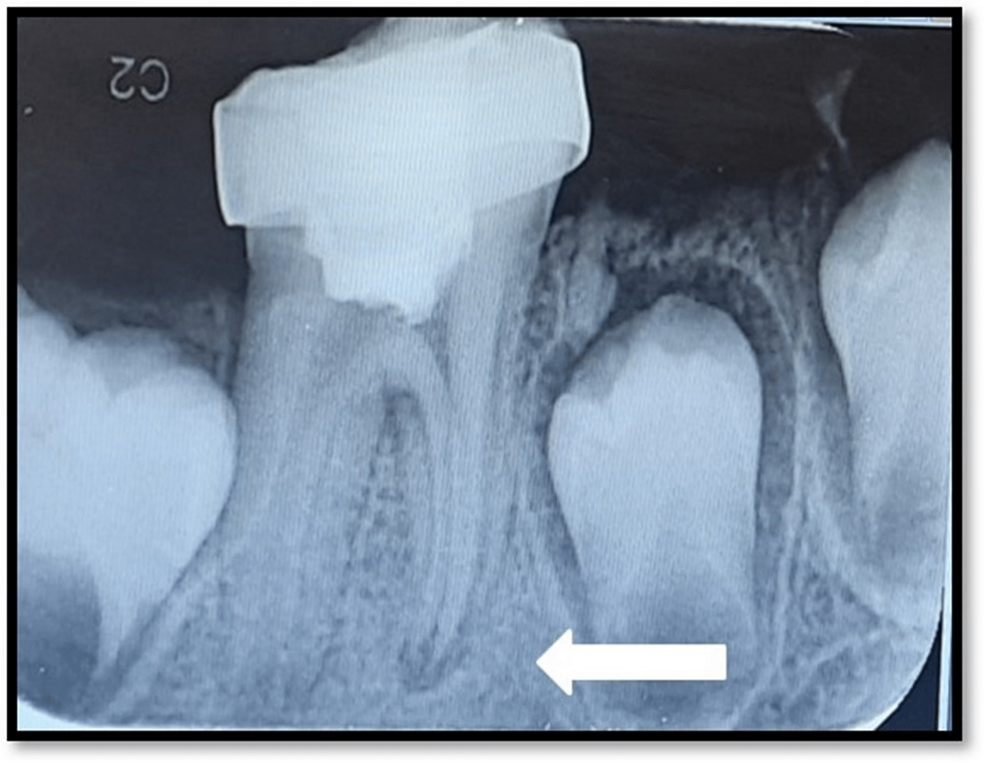
Post-operative radiograph of 6 months follow up. An increase in the root apex formation could be seen with the mandibular right first molar at 6 months follow up (White arrow).

The patient was called back every three months for a radiographic examination and clinical sign and symptom evaluation. There was no discomfort or pain to percussion or palpation, and it was asymptomatic and did not react to cold or electric pulp tester pulp sensibility testing. An increase in the root apex formation could be seen at 6 months follow-up (Figure 4).

## Discussion

Pulp revascularization was the best course of action to preserve and aid the development of the teeth and encourage root formation. According to the updated "American association of endodontics (AAE)" guidelines, the main objective of regenerative endodontic procedures is to treat apical periodontitis [9]. The secondary objective is, by the standards, to improve root wall thickness or length. Regaining a favorable response to pulp testing is the secondary objective. Although desired, secondary and tertiary goals may not be necessary to judge clinical effectiveness [9]. Properly disinfecting root canals during regenerative endodontic treatment can be difficult and contentious.

On the other hand, teeth with an immature apex have larger root canals, making it easier for antimicrobial agents to enter the root canal system and move into the periapical region. It is critical to prevent relatively toxic antimicrobial medications from entering the periapical tissues because they may harm stem cells and vasculature crucial for the regeneration process.1.5% NaOCl was used to irrigate the root canal system before being finished with regular saline [10]. The antibiotic dressing was taken off the canal on the subsequent visit using regular saline. To provide growth factors obtained from dentin, 17% EDTA was administered as the final irrigant after that [11,12]. NaOCl has been demonstrated to be toxic to stem cells from the apical papilla (SCAP) and to prevent stem cells from adhering to dentin surfaces at half- or full-strength concentrations (3% and 6%, respectively) [11,13].

Additionally, it was discovered that the most hazardous irritant for SCAP was 2% CHX. It is important to note that full-strength NaOCl has been utilized for irrigation in several published successful revascularization cases, at least during the first appointment [6,14]. According to earlier investigations, the application of 17% EDTA considerably improved the adhesion of freshly generated mineralized tissues to dentinal canal walls [12]. Growth factors are released from the root canal dentinal walls after EDTA irrigation. A lower concentration of NaOCl and the addition of EDTA could have enhanced the treatment's results.

A potential scaffold for pulp revascularization treatments, platelet-rich fibrin controls inflammatory responses and promotes growth and regeneration. It also serves as a fantastic matrix to facilitate MTA placement [8].

A freshly extracted PRF membrane was inserted into the canal space as part of the deliberate revascularization technique. More evidence must be given regarding its handling and positioning in the root canal space. According to clinical experience, progressive deployment of PRF fragments is more practical than complete membrane placement. To achieve a coronal seal, MTA has proven to have exceptional sealing abilities and was positioned immediately above PRF [15]

PRF delivered growth factors into the cleaned root canal space, acting as a bioscaffold. It is known that mesenchymal stem cells from the apical papilla are present in platelets, and this might have caused them to spread out from the periapical area and produce a matrix in the canal space. Thus, PRF enhanced the growth of new hard and soft tissue within the canal area by serving as a repository of tissue healing elements [8]. Effective root canal system disinfection is essential for revascularization/revitalization therapy to be successful. The pulp-periapical tissue complex will not regenerate or heal if the root canal infection is left untreated [16].

## Conclusions

Pulp revascularization is suggested as an alternative to apexification for developing teeth in endodontic treatment for irreversible pulpitis and pulp necrosis, whether or not associated with the periapical disease. It is a technically straightforward procedure with favorable results since, in contrast to apexification, it encourages dentin wall thickness and apical closure and prevents tooth weakening.
